# Oxidation of MBE-Grown ZnTe and ZnTe/Zn Nanowires and Their Structural Properties

**DOI:** 10.3390/ma14185252

**Published:** 2021-09-13

**Authors:** Katarzyna Gas, Slawomir Kret, Wojciech Zaleszczyk, Eliana Kamińska, Maciej Sawicki, Tomasz Wojtowicz, Wojciech Szuszkiewicz

**Affiliations:** 1 Institute of Physics, Polish Academy of Sciences, Aleja Lotnikow 32/46, PL-02668 Warsaw, Poland; kret@ifpan.edu.pl (S.K.); wzal@MagTop.ifpan.edu.pl (W.Z.); mikes@ifpan.edu.pl (M.S.); szusz@ifpan.edu.pl (W.S.); 2 International Research Centre MagTop, Institute of Physics, Polish Academy of Sciences, Aleja Lotnikow 32/46, PL-02668 Warsaw, Poland; wojto@MagTop.ifpan.edu.pl; 3 Institute of High Pressure Physics Unipress, Al. Prymasa Tysiaclecia 98, PL-01142 Warsaw, Poland; ekaminska@unipress.waw.pl; 4 Institute of Physics, College of Natural Sciences, University of Rzeszow, S. Pigonia 1, PL-35310 Rzeszow, Poland

**Keywords:** nanowires, ZnTe, ZnO, oxidation, core/shell structures, Raman scattering, transmission electron microscopy, structural analysis, semiconductors

## Abstract

Results of comparative structural characterization of bare and Zn-covered ZnTe nanowires (NWs) before and after thermal oxidation at 300 °C are presented. Scanning electron microscopy, energy-dispersive X-ray spectroscopy, high-resolution transmission electron microscopy, and Raman scattering not only unambiguously confirm the conversion of the outer layer of the NWs into ZnO, but also demonstrate the influence of the oxidation process on the structure of the inner part of the NWs. Our study shows that the morphology of the resulting ZnO can be improved by the deposition of thin Zn shells on the bare ZnTe NWs prior to the oxidation. The oxidation of bare ZnTe NWs results in the formation of separated ZnO nanocrystals which decorate crystalline Te cores of the NWs. In the case of Zn-covered NWs, uniform ZnO shells are formed, however they are of a fine-crystalline structure or partially amorphous. Our study provides an important insight into the details of the oxidation processes of ZnTe nanostructures, which could be of importance for the preparation and performance of ZnTe based nano-devices operating under normal atmospheric conditions and at elevated temperatures.

## 1. Introduction

One-dimensional (1D) semiconductor nanostructures, such as nanowires (NWs), nanotubes, nanorods, and others, have attracted a great deal of attention due to their potential applications as building blocks in electronic and optoelectronic nanodevices [1]. There are two major approaches to the fabrication of these 1D structures. The “top-down” approach uses nano-lithography followed by a suitable chemical etching. In the “bottom-up” approach the NWs are assembled already in the form of nanometer-sized columns using various appropriate growth modes. In comparison to their bulk counterparts, the main advantages of the latter approach include the possibility of growth on amorphous substrates [2,3], the ability to combine materials with a strong lattice mismatch [4], or a possibility of forcing the given material of interest to adopt an unusual crystallographic structure [5]. The “bottom-up” path allows for further *in situ* material engineering such as an overgrowth of the so-obtained NWs by yet another material to obtain core-shell or even core-multishell structures. Zinc blende GaAs/(Ga,Mn)As core-shell NWs [6] and wurtzite (Ga,Mn)As nanotubes [7,8] are among the best examples of this latter possibility. The core-shell approach has been proven successful in activation of the band gap emission in ZnTe NWs [9], or via the adequate strain engineering, in achieving a huge anisotropy in the spin splitting of the excitonic photoluminescence [10], or in an about 100 K enlargement of the Curie temperature of MnAs nanocrystals stabilized in an adequate core-multishell structure [11].

Among many materials, zinc oxideis particularly intensively researched due to its wide direct-gap of 3.37 eV and a large exciton binding energy of 60 meV. The non-intentionally doped ZnO usually exhibits *n*-type conductivity and is difficult to convert to *p*-type. Contrary to ZnO, ZnTe (direct-band gap of 2.25 eV) can be relatively easily highly *p*-type doped and can form a *p*-*n* junction with ZnO. Due to the type-II band alignment and the spatial proximity of the band edge wavefunctions to the interface between these two materials [12,13], ZnTe/ZnO heterostructures can absorb light from near ultraviolet to infrared region. It has been shown that ZnO/ZnTe heterostructures in 1D core/shell geometry can be used in photovoltaics [12,13,14,15], photocatalysis [16,17], and photodetectors [18,19].

There exist many approaches to fabrication of 1D ZnTe/ZnO heterostructures, (most of them combine two different growth techniques), such as chemical vapor deposition (CVD) growth of ZnO NWs, followed by ZnTe coverage by metal-organic CVD [14]; molecular beam epitaxy (MBE) growth of ZnTe NWs, followed by ZnO coverage by the atomic layer deposition [20,21]; vapor phase transport growth of ZnO nanorods followed by ZnTe coverage by RF magnetron sputtering [19]; electrochemical deposition approach for production both, the core and the shell compounds [17] or a partial oxidation of ZnTe NWs [18,22].

The method of the oxidation of ZnTe has been proved highly successful in obtaining planar *p*-type ZnO and homogeneous dilute magnetic semiconductor (Zn,Mn)O [23,24]. In this case the starting ZnTe epilayers were heavily *p*-type or Mn doped, respectively. Following the successes of this method, we have been motivated to apply this approach to obtain high-quality ZnO NWs. However, it is not possible to follow directly in the footsteps of the work in references [23,24]. The major constrain here is a substantially lower melting temperature of the free-standing nanostructures than the melting temperature of the epilayers of the same material, which is thermally anchored to a bulky substrate. The melting point of bulk ZnTe is about 1300 °C and the optimum temperature for planar *p*-ZnTe conversion was only 600–650 °C [23,24], a mere half of the melting one. Unfortunately, the free-standing ZnTe NWs melt already below 500 °C and their actual melting point decreases substantially with the reduction of their diameter, assuming values as low as 450 °C for NWs, which are 100 nm in diameter and 430 °C for 50 nm ones [18]. Conversely, it was shown that oxidation processes carried out at 250 °C [18] and 260 °C [25] resulted in ZnO layers of a rather mediocre quality. It is, therefore, of a great interest to attempt the oxidation process at higher temperatures, while still sufficiently low enough to stay safely away from the very low melting temperature of very thin NWs.

In this article, we report the results of our studies of the low-temperature oxidation of MBE-grown ZnTe-based nanowires. Following the material constrains described above, we perform the oxidation at *T*_O_ = 300 °C. The NWs studied here are subjected to a whole suite of structure characterization techniques, including scanning electron microscopy (SEM), energy-dispersive X-ray spectroscopy (EDX), high-resolution transmission electron microscopy (HRTEM), and room temperature micro-Raman scattering (μ-RS). Our characterization effort confirms the formation of ZnO, but the material is found only in the outer layer of the oxidation-modified NWs. After finding that ZnO assumes the form of separated nanocrystals decorating the NWs’ cores, we decided to check whether an additional Zn coating of the ZnTe NWs prior to the oxidation could improve the ZnO morphology. In fact, it does. ZnO shells that are obtained upon the oxidation of the Zn coated NWs assume a fine-crystalline or amorphous structure. In both cases, instead of the complete transformation of the whole ZnTe material into ZnO, the applied thermal processing leads to NWs assuming either ZnTe/Te/ZnO core/shell structure form, or after sufficiently long oxidation time, the form of Te NWs covered with ZnO.

## 2. Materials and Methods

The base ZnTe NWs are grown on GaAs (111)-oriented substrates by molecular beam epitaxy (MBE) at 400 °C employing a gold-catalyzed vapor-liquid-solid (VLS) mechanism, following the method detailed in references [26,27]. As documented therein, this method produces NWs of high structural quality; in particular, no signs of Te precipitates are brought about by X-ray diffraction (XRD), cf. Figure 3 from reference [27], Figure 2 in [28], and Figure 2 in [20]. This fact is of a great importance for the study reported here. We grow two separate samples of ZnTe NWs under nominally the same conditions. The first sample has been taken out from the growth chamber without any additional modifications. The NWs from the second growth are additionally in situ covered by a thin layer of Zn using the same atomic Zn flux as for the growth of the NWs. This process took 15 min at the substrate temperature reduced to 350 °C. In order to increase the uniformity of the Zn coverage, the sample has been rotated during the metal deposition.

Four hours long oxidation process of both types of the NWs has been performed in a separate furnace at 300 °C in the flowing O_2_ gas. The NWs’ surface morphology is examined by Zeiss Auriga SEM equipped with an EDX system from Bruker AXS (Quantax). Morphology and structure of the NWs have been investigated using FEI Titan 80–300 TEM operating at 300 kV with a spherical aberration corrector of objective lenses in the HRTEM mode. For TEM investigation the NWs have been mechanically transferred onto copper grids covered with holey carbon films, enabling imaging of the entire NWs. The room temperature μ-RS measurements are performed using a Renishaw InVia Raman system with a spectral resolution close to 1 cm^−1^. The He-Cd 325 nm laser line and the 514 nm line emitted by Ar^+^ laser are applied for the excitation. The spectra are collected using a microscope with ×10 objective in the case of the 325 nm laser excitation (yielding the diameter of the spot size on the sample surface of about 5 μm) and with ×50 objective when the 514 nm laser excitation has been used (here the diameter of the spot size on the sample surface is about 1 μm). In order to eliminate spurious signals originating from the polycrystalline Zn-Te-like layer deposited on the GaAs substrate between the NWs, some NWs are transferred from the original substrate onto Si or GaAs wafers for the EDX and μ-RS measurements.

## 3. Results and Discussion

**Figure 1:**
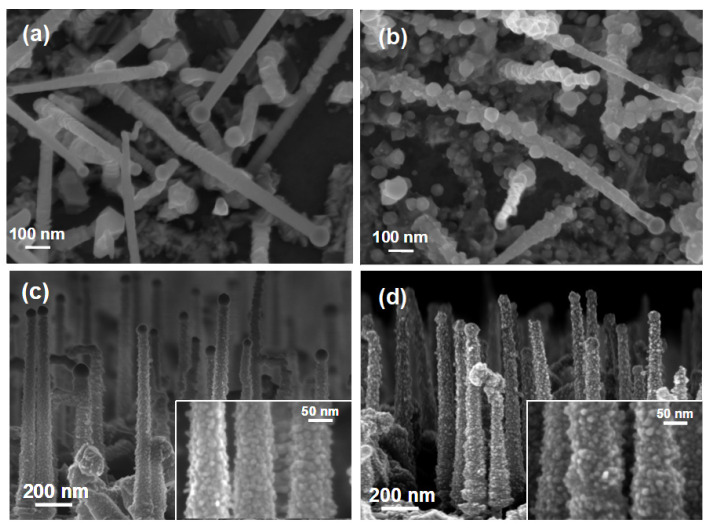
Scanning electron microscopy images of (**a**) as-grown ZnTe NWs; (**b**) oxidized ZnTe NWs; (**c**) as-grown ZnTe/Zn NWs; (**d**) oxidized ZnTe/Zn NWs.

**Figure 2:**
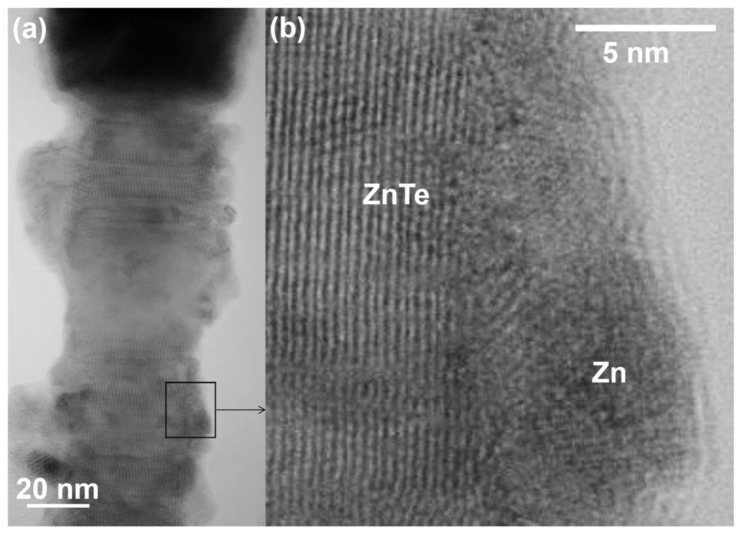
Transmission electron microscopy images of (**a**) part of as-grown Zn-covered ZnTe NW; (**b**) the blown-up section indicated in panel (**a**). This panel indicates a high crystallographic quality of ZnTe NW’s core and a polycrystalline Zn shell.

Figure 1 collects representative SEM images of the bare ZnTe (top panels, bird’s eye view) and Zn covered ZnTe NWs (bottom panels, side view). The as-grown material is depicted in the left panels, whereas the morphology of the corresponding NWs after the oxidation is shown in the right panels. The NWs are typically 1–2 μm long and their diameters exhibit a distribution with an average of about 50 nm (as established in their central parts). Small droplets of the catalyst are seen on the NWs’ tips in all of the images. Numerous stacking faults, typical for this growth method [27] are seen in the lower parts of ZnTe NWs (Figure 1a). The Zn coating uniformly decorates the surface of the bare ZnTe NWs assuming a form of densely located small Zn nanocrystals (Figure 1c), resulting in an increase of the NW diameter by about 10 nm. The high quality of ZnTe cores of both types of the NW’s studied here are presented in Figure 2, which shows exemplary TEM images of an as-grown Zn covered ZnTe NW. Panel (b) of this Figure presents a blown-up part of the image in panel (a), which exemplifies lattice fringes characteristic for ZnTe crystal with a regular structure. This panel also clearly indicates the polycrystalline structure of the Zn shell. Some stacking faults perpendicular to the growth direction [111] are clearly resolved.

The 4 h long oxidation at 300 °C changes the morphology of both types of the NWs. By far, more pronounced changes are seen for the bare ZnTe NWs, where numerous nanocrystals, up to 60 nm in diameter, appear on their surface (Figure 1b). It is also worth noting that after the oxidation, the stacking faults defects, which were present in the lower parts of ZnTe NWs, have disappeared. In the case of the Zn-covered NWs, the analogous oxidation process does not lead to such significant changes in the morphology. Here, only a slight increase in the sizes of the crystallites building the NWs’ shells and an increase in NWs’ diameters to 80–90 nm can be noticed (as presented in the insets in Figure 1c,d. Moreover, some changes in the NWs’ surface morphology can be seen in the region of their tips.

**Figure 3:**
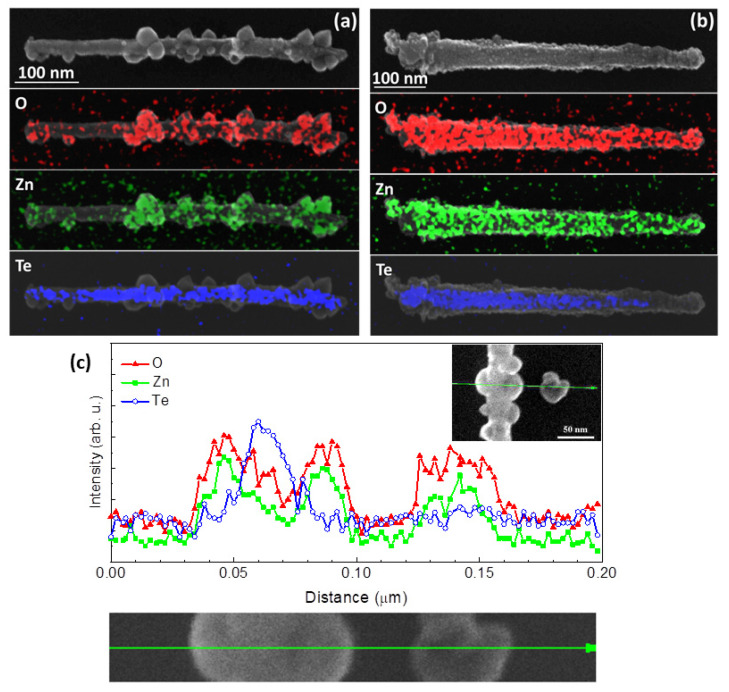
Scanning electron microscopy micrographs of oxidized (**a**) ZnTe and (**b**) ZnTe/Zn NW and related to them energy dispersive X-ray (EDX) spectroscopy distribution maps of O, Zn, and Te. (**c**) Transverse EDX line scan running across the oxidized ZnTe NW and one of its loose crystallites. Data corresponding to O, Zn, and Te yields are plotted as red triangles, green squares, and blue circles, respectively. The experimental configuration is depicted in two insets. Below and at the top right corner of the main graph.

The analysis of the elemental distribution within the NWs after the oxidation process, carried out by the EDX method is presented in Figure 3a,b. In particular, we find that in the case of the oxidized bare ZnTe NWs (Figure 2a) only the large nanocrystals decorating the surface of the NWs contain Zn and O. The rest of the NWs’ structure has been substantially devoid of Zn. Essentially, the same chemical structure of the oxidized ZnTe NWs is presented in Figure 3c. In particular, the strongest Te signal is registered at the center of the NWs, while the separated crystallite is composed exclusively of Zn and O. The EDX data unequivocally state that upon the oxidation these NWs have lost their original ZnTe nature and transformed into Te inner cores decorated with ZnO nanocrystals. The results of EDX analysis of the oxidized ZnTe/Zn NWs are exemplified in Figure 3b. We find that in this case Zn and O are distributed uniformly along the entire length and circumference of the NW, whereas Te is present only in its central part. This indicates the formation of a ZnO shell encircling the Te-rich core.

**Figure 4:**
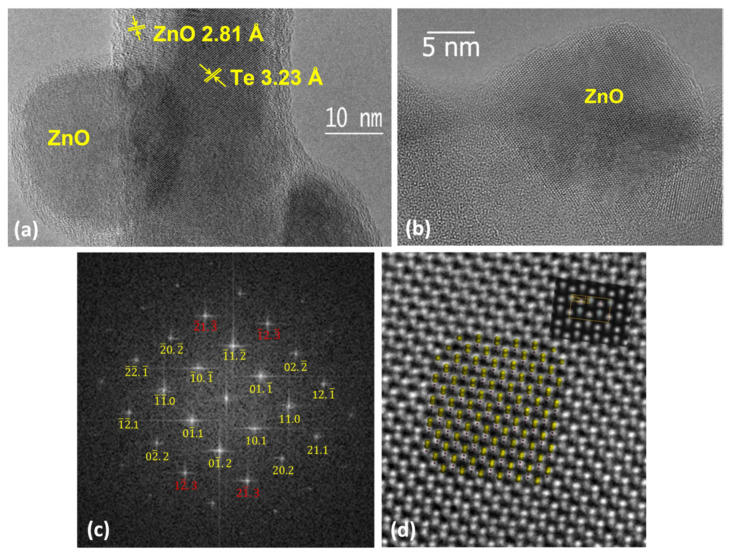
The TEM analysis of the structure of the oxidized ZnTe NW, similar to these presented in Figure 1b and Figure 2a. Panels (**a**,**b**) zoom in these areas of the NWs in which the large crystallites are formed. The distance markers in (a) represent the dominant interlayer spacings in the core and the edge parts of the NWs. (**c**) 2D Fourier transform obtained for ZnO crystallite seen in (**b**). (**d**) High-resolution TEM image taken in $\left[\bar{1}1.1\right]$ zone axis of a small fragment of the same crystallite. The simulated image (right top inset) and ball model of projection of ZnO wurtzite structure are superimposed.

The structural analysis of a representative oxidized bare ZnTe NW is presented in Figure 4. The different interplanar spacings in the central part of the NW and near its edge, as marked in Figure 4a, indicate a somewhat composite structure of the ZnTe NWs after the oxidation. The central part of these NWs is composed of Te (the interplanar spacing of 3.23 Å at the center of the NW corresponds very well to the (10.1) lattice plane spacing of hexagonal tellurium), whereas the interplanar spacing of 2.81 Å found close to the edge matches the (10.0) spacing of ZnO. These NWs are decorated sparsely by relatively large ZnO crystallites. Zoom into one of such ZnO crystallites is presented in Figure 4b. The 2D Fourier transform of this image is given in Figure 4c, confirming its wurtzite structure. Detailed analysis carried out using the methodology based on the comparison of simulated electron-microscopic images with experimental high-resolution images further confirms that these crystallites are made of wurtzite ZnO. The positions of the Bragg peaks on the Fourier transform from Figure 4c are fitted to the simulated electron diffraction, assuming that the electron beam is parallel to the direction $\left[\bar{1}1.1\right]$ (Figure 4c). Simultaneously, simulations of HRTEM images for wurtzite ZnO observed in the $\left[\bar{1}1.1\right]$ direction were performed and a good agreement was obtained between the experimental background image and the simulated images, as presented in the inset located in the upper right corner of Figure 4d. The local intensity peaks in the HRTEM image coincide with the Zn and O bi-columns. The maximum values corresponding to the zinc bi-columns show higher intensity both in the simulations and in the experimental images.

**Figure 5:**
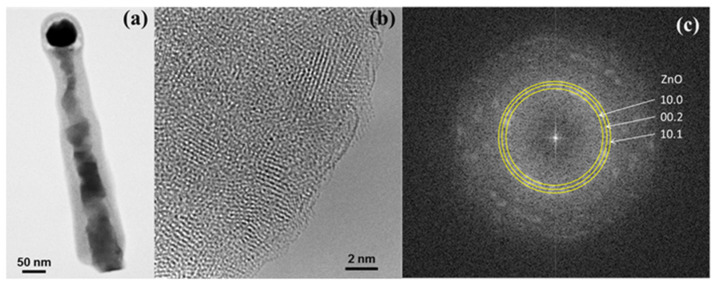
The TEM structure analysis of oxidized ZnTe/Zn nanowire, similar to these presented in Figure 1d and Figure 2b. Panel (**a**) presents a general image of such NW, panel (**b**) shows a high resolution close up of the NW outer shell. (**c**) Modulus of the two-dimensional Furrier transform in logarithmic scale obtained for that part of the NW that is seen in (**b**). The Debye–Scherrer rings indicated in the panel correspond to ZnO crystal planes distances in the nanocrystallites.

Exemplary results of the structural characterization performed on an oxidized ZnTe/Zn NW are presented in Figure 5. In this case, a clear core-shell structure of the NW has been confirmed (Figure 5a). The total diameter of this particular NW measured at a half-height is approximately 90 nm, while the thickness of the shell is approximately 25 nm. Fine details of the structure at the edge of the shell of the NW are presented in the HRTEM image included in Figure 5b. This image indicates an ultrafine grained ZnO with the grain size in the range between 1 and 2.5 nm. Further information about the structure is obtained from the two-dimensional Furrier transform (2D-FFT) of this image. This 2D-FFT is presented in panel (c). The lattice planes of ZnO nanocrystallites, which are parallel to the electron beam direction are represented in the Fourier space as individual broad Bragg peaks due to diffraction size-like effect (there are only a handful of parallel planes and they are of a small size in every nanocrystal). These peaks are arranged on the Debye–Scherrer rings corresponding to crystal planes distances of ZnO nanocrystallites. The procedure also reveals a presence of an amorphous material without well-defined long-range ordering. It contributes to a relatively high intensity of the background of the 2D-FFT in comparison to the intensity of the Bragg peaks. 

We can summarize our TEM studies by stating that the results of the oxidation are different for the bare ZnTe NWs than for those coated with Zn. While, in case of the bare ZnTe NWs, some large separated ZnO wurtzite nanocrystals are formed on the Te-rich core, the covering of ZnTe by Zn leads to the formation of the more homogeneous ZnO shell, but with a much more mediocre crystallographic quality.

**Figure 6:**
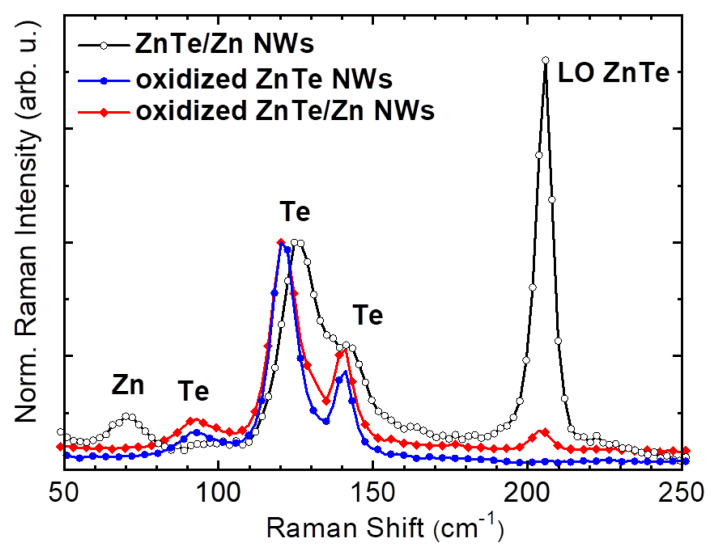
Raman scattering spectra of unprocessed (black circles) and oxidized ZnTe (blue points) and ZnTe/Zn (red diamonds) nanowires. These spectra are collected at ZnTe resonant conditions (λ = 514 nm) and normalized to the Te feature at about 120 cm^−1^.

Complementary information on the structural changes taking place in ZnTe NWs as a result of the oxidation process is obtained from Raman scattering. The representative Raman spectra acquired for both types of NWs transferred onto Si wafers are presented in Figure 6. These spectra were collected using the 514 nm laser line having photon energy (2.41 eV) that is close to the band gap energy of ZnTe. All the spectra are normalized with respect to the feature that is present at about 120 cm^−1^, which we attribute to tellurium, as explained below. We start from the unoxidized ZnTe/Zn NWs (black circles) for which the dominant feature is a strong ZnTe LO-phonon peak near 206 cm^−1^ [29]. Also, three LO-phonon replicas at multiples of this frequency are observed (not shown here). This spectrum clearly reveals the presence of Te (three features between 85 and 150 cm^−1^), which is ubiquitously detected in tellurium compounds [30,31]. On the other hand, a very fine synchrotron-based XRD studies had not even hinted at a presence of Te precipitates in equivalent NWs [20,27,28]. This apparent discrepancy arises from the very high cross-section of Te in the Raman spectroscopy performed under the visible light excitation [30], yielding large responses even for truly miniscule quantities. The feature at the frequency near 70 cm^−1^ can be attributed to the scattering on Zn optical phonon [32]. However, this line is located about 2 cm^−1^ lower in energy and its linewidth is higher than that for the polycrystalline Zn samples [32]. We explain this discrepancy by the size effect. Overall, these findings confirm the expected chemical structure of the NWs prior to the oxidation. In the Raman spectrum obtained for the unoxidized bare ZnTe NWs (not shown here), the Zn-related low-energy peak is absent, while the rest of the spectrum is qualitatively the same.

Full symbols in Figure 6 mark the Raman spectra acquired for the same excitation energy for both types of NWs after the oxidation. The spectra were normalized to one of the Te features at around 120 cm^−1^. The most important observation is the dramatic reduction of the amplitude of the ZnTe LO phonon line. Only its weak trace can be found for the oxidized ZnTe/Zn NWs and it is completely absent in the spectrum corresponding to the oxidized bare ZnTe NWs. Moreover, after the oxidation, the whole Te-related structure between 85 and 150 cm^−1^ has become sharper with the new positions of its most prominent features at 92, 121, and 141 cm^−1^, corresponding now very well to E, A_1_, and E_1_ phonon modes of the crystalline hexagonal Te, respectively [33]. The differences in the shape and position of these complex Te-related structures for the unprocessed and oxidized samples indicate that Te assumes different forms in these two types of samples [34]. It is far more amorphous in the non-oxidized NWs, whereas it acquires substantially more crystalline structure after annealing in oxygen. For the oxidized ZnTe/Zn NWs (red diamonds), the Zn-related peak is missing, indicating that the zinc layer surrounding the ZnTe NWs has been completely oxidized.

**Figure 7:**
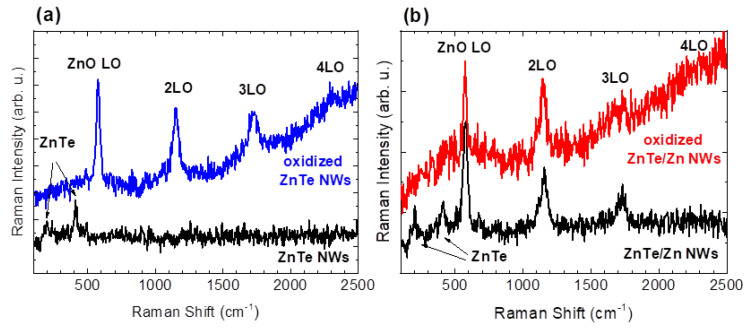
Raman scattering spectra of unprocessed (bottom black curves) and oxidized NWs for (**a**) bare ZnTe NWs (upper blue curve) and (**b**) Zn-covered ZnTe NWs (upper red curve). These spectra are collected at ZnO resonant conditions (λ = 325 nm).

Completely different Raman spectra are obtained when the NWs are excited with a 325 nm laser line, having photon with energy close to that of the electronic interband transition of the wurtzite ZnO. Figure 7 compares such spectra taken on the unprocessed (bottom curves) and oxidized NWs (upper curves in both panels (a) and (b) for the ZnTe and ZnTe/Zn NWs, respectively). These two pairs of spectra are quite similar, and in both cases a strong ZnO LO line (at 576 cm^−1^ [35]) and its replicas dominate the spectra collected after the oxidation. The other common feature in these panels is the presence of two peaks at about 205 cm^−1^ and 410 cm^−1^. These frequencies correspond to the LO phonon mode of ZnTe and its first overtone, respectively, and are no longer visible after the oxidation. The main difference between these panels is seen in the bottom spectra acquired for the unprocessed materials. While the left spectrum obtained for the ZnTe NWs remains featureless above 500 cm^−1^, a full train of the ZnO LO line and its two replicas present in this spectral range indicate that partial oxidation of the Zn shells of the ZnTe/Zn NWs takes place already after taking the NWs into the ambient, i.e., without subjecting the NWs to a dedicated oxidation process.

We can summarize our characterization effort by stating that the oxidation of ZnTe NWs performed at temperature of 300 °C yields different results depending on whether the bare ZnTe or Zn-covered ZnTe NWs are processed. In the case of ZnTe NWs, our findings confirm earlier findings of Lu et al. [25] (*T*_O_ = 260 °C) that for the oxidation performed substantially below 600 °C (i.e., below the optimum temperature for ZnTe → ZnO transformation [23,24]), instead of sharp ZnTe/ZnO interfaces—a ZnTe/Te/ZnO sequence is obtained. As argued in [25] the dominant mechanism at such low temperatures is the out-diffusion of Zn atoms towards the NWs’ surface and formation of ZnO crystals by combining with atmospheric oxygen. The formation of ZnO crystals on the surface is more likely than formation of TeO_2_ ones, due to the lower energy cost of formation of the former (−323.9 kJ/mol) than of the latter (−347.8 kJ/mol) [25]. Upon sufficiently long oxidation the remaining Te forms a semimetallic core of the converted NWs.

It is an interesting fact that while the out-diffusion of Zn from ZnTe has become a well-established experimental fact [25], the microscopic origin of this process remains yet to be revealed. Indeed, although Zn species are 5 to 6 orders of magnitude more mobile than the Te ones in the ZnTe lattice [36], the low magnitudes of the corresponding diffusion constants at around 300 °C imply the chemical stability of ZnTe at our *T*_O_. However, the same study [25] clearly pointed out that this was only a skin effect, which indicates the mechanism is operational only at close proximity to the free surface of the material. More accurately, it operates only in a close proximity to a dense pocket of charged surface states. We postulate, therefore, that this is the combined potential of these charged states that electrostatically drive Zn from their nesting lattice sites at elevated temperatures. While this is only a skin effect in bulk ZnTe crystals, so optical efficiencies of bulk ZnTe crystals are hardly changed, the whole bodies of only tens of nanometer thin ZnTe NWs are nothing more than the surface-proximity-regions. Therefore, such an electrostatic potential can draw nearly all of Zn from the NWs, quenching completely the characteristic luminescence of ZnTe, despite the excellent crystalline properties of these NWs [26]. In fact, it took some technological attempts to regain the photoluminescence from ZnTe NWs. The characteristic, intense emission in the near band edge energy region of ZnTe had been successfully recovered in ZnTe NWs only after an *in situ* coverage of the bare ZnTe NWs by a wider band-gap relative ternary compound (Zn, Mg)Te [37]. The authors of this report directly linked the effect of the NWs’ coverage to the passivation of charged surface states. The same approach had been shown very effective in III-V NWs [38]. In further support of our conjecture, we note that the presence of the electrostatic potential generated by the surface states had been also identified to be the leading mechanism responsible for the existence of the gradient of Mn interstitials in (Ga,Mn)As [39]. Importantly, this gradient forms during the MBE growth at temperatures between 200 and 300 °C, i.e., comparable to our *T*_O_ = 300 °C, and the Mn depth profile stabilizes after cooling down the sample to the ambient temperature.

Interestingly, in their study, Lu et al. [34] found ZnO in the form of randomly oriented crystallites with diameters in the range of a few nanometers, as well as in an amorphous phase. Both uniformly covering the free flat surface of the bulk ZnTe. The ZnO nanocrystals formed in our study are much larger, up to 60 nm in diameter, and they only sparsely decorate the Te cylindrical cores. We assign this sizable difference to a much stronger curvature of the surface of the NWs. There, as discussed previously, numerous broken bonds and variously configured dimers are expected. This sizably enlarges the surface mobility of Zn atoms, creating conditions required for the Stransky–Krastanov growth mode [40], extensively used to promote quantum dots formation during the epitaxial growth [41]. Importantly, a several monolayer thin ZnO wetting layer present between the large NCs is resolved in Figure 4a. By the same token, the additional Zn coverage of the NWs heals their troubled free surface and so reduces the surface mobility of Zn. This, in turn, promotes far more even surface coverage by the ZnO material, precluding the formation of separated islands. This uniform ZnO layer is, however, of a mediocre quality, partially amorphous, partially fine crystalline. Conversely, even in this case, the complete transformation into pure ZnO has not taken place. After the oxidation, as for the bare ZnTe NWs, the cores of Zn/ZnTe NWs are composed predominantly of Te. Again, it is the result of the decomposition of the core ZnTe and out-diffusion of Zn.

## 4. Conclusions

The possibility of the transformation of ZnTe nanowires into ZnO ones upon the low-temperature oxidation process at 300 °C has been studied. All the characterization methods applied here indicate the formation of ZnO in the outer layer of the oxidation-modified NWs. However, the actual form of ZnO depends on the initial state of the NWs surface. The presence of the additional Zn coverage results in a relatively smooth ZnO shell, but only of an amorphous or a fine-crystalline form, whereas in the case of the oxidation of bare ZnTe NWs only separated and relatively large ZnO nanocrystals form. In this sense, the morphology of the resulting ZnO can be improved by the deposition of thin Zn shells on the bare ZnTe NWs prior to the oxidation. Interestingly, the thermal processing of both types of NWs yields a sizable enrichment in Te of the NWs cores. This Zn-outdiffusion driven process can yield Te-core based structures, which have become a great interest recently [42]. A process rooted in the electrostatic attraction of Zn from the bulk of the NWs by charged surface states has been suggested to explain the out-diffusion of Zn from the inner ZnTe.

It can be, therefore, concluded that the low-temperature oxidation of ZnTe and Zn covered ZnTe NWs does not seem to be a promising method of obtaining high-quality ZnO-based nanostructures, since the oxidation process leads rather to the formation of a ZnTe/Te/ZnO sequence, which upon sufficiently long oxidation time turns to Te/ZnO one. In that aspect, this approach can be envisaged as a possible path for manufacturing low-dimensional Te nanostructures instead of pure ZnO NWs and/or ZnO/ZnTe heterojunctions. Other important findings of this study are summarized in the following two points. Firstly, our effort indicates that it is very unlikely to form a homogeneous and good quality ZnO/ZnTe heterojunction by the oxidation technique at the conditions exercised in this study. Here only Te/ZnTe and Te/ZnO interfaces have been obtained. However, it remains possible that further and wider ranging studies could end up in realization of structures of a much superior structural quality. One of the examples can be a multi-step oxidation in progressively increasing temperatures. Secondly, in a more general view, our research sheds some light on the reasons behind the aging degradation of the performance of ZnTe based nano-devices operating under normal atmospheric conditions and at elevated temperatures.

## Data Availability

Not applicable.
